# Causal relationship between green tea intake and gastrointestinal disorders: a two-sample Mendelian randomization study

**DOI:** 10.3389/fnut.2024.1426779

**Published:** 2024-09-20

**Authors:** Chan Chen, Yifei Lin, Jinni Xu, Qingquan Chen, Jing Huang

**Affiliations:** ^1^Fujian Vocational College of Agricultural, Fuzhou, China; ^2^Fujian Medical University, Fuzhou, China

**Keywords:** green tea intake, Mendelian randomization, gastrointestinal disorders, association study, causal relationship

## Abstract

**Background:**

The precise association between green tea intake and gastrointestinal disorders remains controversial. This study aimed to investigate the potential causal association between green tea intake and gastrointestinal disorders through a two-sample Mendelian randomization (MR) study.

**Methods:**

Utilizing publicly accessible data from genome-wide association studies (GWAS), we identified SNPs strongly linked with the study variables from multiple large databases to serve as instrumental variables (IVs). MR analyses were executed utilizing the inverse variance weighting (IVW) method, with the resultant effect estimates serving as the primary outcome measure. In addition, a multivariate MR design was performed to adjust for smoking and alcohol consumption. To ensure the robustness of our findings, a series of sensitivity analyses were conducted to assess reliability.

**Results:**

Univariable MR analysis revealed suggestive associations between green tea intake and gastroesophageal reflux (OR = 0.9950, 95% CI 0.9900–1.0000, *p*_IVW_ = 0.047), diverticulosis (OR = 0.9998, 95% CI 0.9996–1.0000, *p*_IVW_ = 0.030), Crohn’s disease (OR = 1.0001, 95% CI 1.0000–1.0002, *p*_IVW_ = 0.019), and cholangitis was observed (OR = 1.0440, 95% CI 1.0100–1.0790, *p*_IVW_ = 0.011). Multivariate MR analysis indicated after controlling for potential confounders, greater green tea consumption was suggestively associated with the decreased risk of oesophagitis (OR = 0.9667, 95% CI: 0.9405–0.9936, *p*_IVW_ = 0.016) and gastric cancer (OR = 0.9810, 95% CI: 0.9628–0.9996, *p*_IVW_ = 0.046). Nevertheless, multivariate MR analysis also showed that greater green tea consumption was suggestively associated with the increased risk of Crohn’s disease (OR = 1.0001, 95% CI: 1.0000–1.0002, *p*_IVW_ = 0.007). Sensitivity analyses confirmed that these results were reliable.

**Conclusion:**

Our study provides suggestive evidence that genetically predicted green tea intake is causally associated with the risk of oesophagitis, gastric cancer and Crohn’s disease, but a larger GWAS database is needed for validation.

## Introduction

Gastrointestinal disorders afflict a significant portion of the population, with approximately 50 million visits yearly recorded for such conditions (1, 2). As highlighted by Peery et al. (3), these disorders lead to millions of healthcare encounters and hundreds of thousands of deaths annually in the United States, incurring billions of dollars in costs. This category encompasses a diverse array of conditions, including gastroesophageal reflux disease (GERD), diverticular disease, Crohn’s disease, and cholangitis. Gastroesophageal reflux disease (GERD) stands out as one of the most prevalent gastrointestinal disorders, often linked to lifestyle and dietary factors (4). It has also been suggested that beverage consumption may be a potential risk factor for gastroesophageal reflux disease (GERD) in young adults (5).

Meanwhile, diverticulosis, a prevalent condition, is increasingly prevalent in Western societies (6), with dietary habits identified as a contributing risk factor for its complications (7). Crohn’s disease (CD) triggers chronic inflammation within the gastrointestinal tract (8), often leading to malnutrition and heightened morbidity and mortality (9). Additionally, cholangitis, characterized by life-threatening biliary infection, exhibits mortality rates nearing 100% (10). Hence, investigating the potential link between beverage consumption patterns and gastrointestinal disorders holds paramount importance for our everyday health. Tea ranks among the most widely consumed beverages globally and is rich in catechins, natural polyphenolic phytochemicals renowned for their potent antioxidant properties (11, 12). Catechins play a pivotal role in mitigating oxidative stress by effectively inhibiting its excess and facilitating the activation of key antioxidants like glutathione peroxidase (GPO) and glutathione (GSH), thereby curbing oxidative damage within the gastrointestinal tract (12).

Numerous studies have shown that tea consumption may be closely linked to non-malignant digestive diseases (13). For instance, excessive consumption of green tea could pose a risk factor for gastroesophageal reflux disease (GERD) (13), while tea intake appears to have a negative association with the risk of Crohn’s disease (14). Additionally, long-term green tea consumption has demonstrated a preventive effect by reducing the risk of gastric and colorectal cancers (15, 16). Nevertheless, the precise causal relationship between green tea consumption and gastrointestinal diseases remains elusive. Furthermore, limitations such as small sample sizes and potential biases in study population selection may hinder the generalizability of certain findings. Moreover, traditional observational studies often struggle to address the challenges posed by reverse causality and confounding factors.

Mendelian randomization (MR) represents a form of instrumental variable analysis that leverages genetic variations as instrumental variables. MR is a type of instrumental variable analysis, which uses genetic variation as an instrumental variable to measure the association between genetic variation and exposure factors, between genetic variation and disease outcome, and then infer the association between exposure factors and disease outcome, and accordingly explore the causal relationship between exposure and outcome (17). In this study, we intend to investigate the causal relationship between green tea consumption as the exposure and gastrointestinal diseases as the outcome through two-sample Mendelian randomization. Additionally, we aim to explore the varying effects of green tea consumption across different gastrointestinal conditions, to offer novel insights into gastrointestinal disease surveillance and preventive strategies for green tea-consuming populations.

## Methods

### Mendelian randomization study design

**Figure 1:**
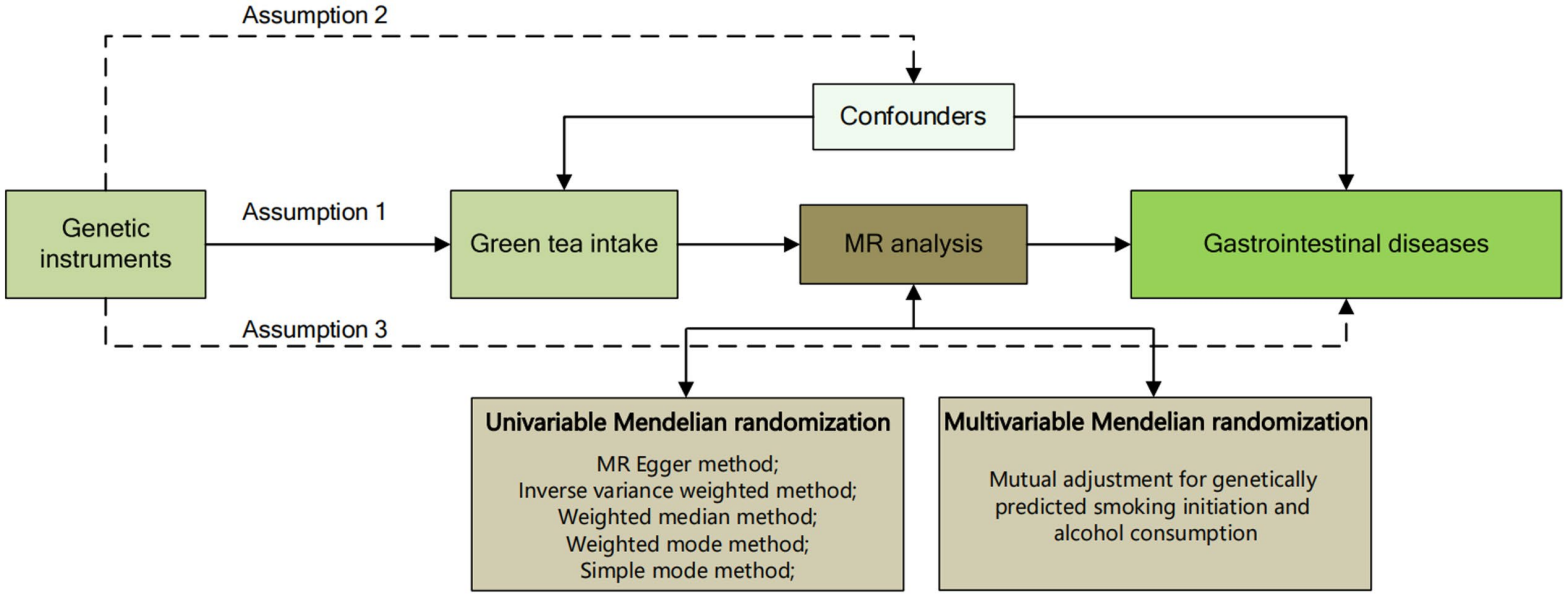
Overview flowchart of assumptions and schematic design. SNPs associated with green tea intake were used as genetic instruments to study the causal effect of green tea intake on gastrointestinal diseases. Lines with arrows indicate that genetic instruments (SNPs) are associated with exposure and can only influence the outcome through exposure. Dashed lines indicate that the genetic tools (SNPs) are not associated with confounders between the results.

MR analysis relies on three fundamental assumptions. Firstly, genetic variants employed as instrumental variables must exhibit robust associations with the exposure under investigation. Secondly, the selected genetic variant should not be linked to any confounding factors. Lastly, the chosen genetic variants should solely influence the risk of the outcome through relevant risk factors (Figure 1). In addition, considering the genetic association of green tea consumption with smoking behavior and alcohol consumption, we further used a multivariate MR design to systematically minimize pleiotropy for both traits. To adhere to ethical guidelines, all original GWAS utilized in this research underwent review and approval by respective institutional review boards. Furthermore, given that this study constitutes a reanalysis of previously collected and published data, no additional ethical approval was deemed necessary.

### Data sources

The pooled data utilized in our study were sourced from genome-wide association studies (GWASs) conducted in European-descent participants from large-scale consortia or studies. For the exposure data, the SNPs associated with green tea intake were identified from 64,949 individuals from UKBiobank. In the original study, green tea intake was assessed as a categorical ordered variable (cups of green tea), with the original questionnaire categorizing tea consumption into 0, 1, 2, 3, 4, 5, and ≥6 cups and each cup corresponds to 250 mL (data field: 100420). For the outcome data, we included 25 gastrointestinal diseases covering upper gastrointestinal diseases, lower gastrointestinal diseases, hepatobiliary and pancreatic diseases, and acute appendicitis. Among them, data for Crohn’s disease and intestinal obstruction were obtained from UKBiobank. The outcome data for hiatus hernia and diverticular disease were obtained from the study of Dönertaş et al. (18). The study of Fairfield et al. (19) provided us with GWAS data for non-alcoholic fatty liver disease. The data of oesophagitis, gastroduodenal ulcer, acute gastritis, chronic gastritis, irritable bowel syndrome, alcoholic liver disease, cholecystitis, cholelithiasis, acute pancreatitis, chronic pancreatitis, and acute appendicitis were from FinnGen database. Gastroesophageal reflux, esophageal cancer, gastric ulcer, gastric cancer, ulcerative colitis, colorectal cancer, cirrhosis, and pancreatic cancer were obtained from the study of Sakaue et al. (20). The outcome data for cholangitis were obtained from the study of Ji et al. (21). Supplementary Table S2 shows the specifics of the GWAS pooled databases employed in our analysis. Details regarding ethical approval and participant informed consent for the GWASs are available in the respective GWAS publications referenced within the manuscript.

### Selection of genetic instruments

During the data extraction phase, single nucleotide polymorphisms (SNPs) from the GWAS data were selected as instrumental variables based on their association with the study factors (*p* < 5 × 10^−8^). Subsequently, SNPs exhibiting linkage disequilibrium were filtered out, employing a correlation threshold parameter set at *r*^2^ ≤ 0.001 and kb ≤10,000. No SNPs associated with potential confounding factors such as smoking initiation and alcohol consumption were identified by the NHGRI-EBI Catalog databases^1^ and LDtrait tool^2^ (22). Ultimately, we identified 21 SNPs significantly linked to green tea intake. Supplementary Table S3 presents the *F*-statistics associated with these SNPs, all of which exceeded 10, thereby ensuring the exclusion of weak instrumental variables and affirming the robust correlation between the instrumental variables and all exposures.

### Two-sample Mendelian randomization analysis

We conducted a two-sample MR analysis to investigate the association between green tea intake and gastrointestinal disorders. The primary MR method employed was inverse-variance weighting (IVW). This method is a standard approach for aggregating MR data, enabling the direct estimation of causal relationships among study subjects through aggregated data (23). Outliers in the IVW linear regression were identified using the Mendelian Randomization of Multiple Phenotype Residual Sums and Outliers (MR-PRESSO) method, and MR estimates were adjusted following outlier removal. Additional complementary methods included IVW (fixed effects), weighted median, weighted mode, and simple mode (Figure 1). Median methods, which necessitate that at least half of the genetic instruments in the pooled data are valid to attain a consistent effect value, encompass unweighted median (simple median), weighted median, and penalized weighted median estimation. Among these, the weighted median combines weighted estimation and median, allowing for the accurate assessment of each genetic variant’s evaluated value based on different causal effect weights (24, 25). Furthermore, if the IVW method yields statistical significance while the other methods do not, the odds ratios (ORs) from the other methods must align in direction with the IVW; otherwise, they are not deemed statistically significant. Moreover, multivariate MR is an extension of univariate MR to jointly detect the causal effects of multiple risk factors (26) and was used to adjust for differences in genetic prediction of green tea consumption, smoking initiation and alcohol consumption (27).

To avoid increasing the risk of Type I errors when performing multiple statistical tests (28), we adjusted the significance level of *p* < 0.002 (0.05/25) using the Bonferroni correction. *p*-values falling above the Bonferroni-corrected threshold but below the conventional significance level (*p* < 0.05) were categorized as suggestive of causal associations.

### Sensitivity analysis

We conducted a sensitivity analysis employing various methods to ascertain the robustness and validity of the results. Heterogeneity in the study results was assessed using the Cochrane *Q*-test, where a *p*-value less than 0.05 indicated the presence of heterogeneity, leading to the utilization of a random effects model; conversely, a *p*-value greater than 0.05 signified the absence of heterogeneity, prompting the use of a fixed effects model. Additionally, the MR-Egger regression intercept term and the MR-PRESSO method were employed to evaluate the horizontal multivariate validity of instrumental variables, with a significance level of *p* < 0.05 suggesting the potential existence of horizontal multivariate validity (29). Horizontal pleiotropy occurs when single nucleotide polymorphisms (SNPs) utilized as instrumental variables influence outcomes through pathways other than the intended exposure. MR Egger regression, a method accounting for potential variable heterogeneity, yields a corrected estimate of the causal effect, with the slope obtained representing this estimate (30). The leave-one-out method involves identifying SNP loci that may impact MR results and sequentially removing non-compliant loci to mitigate the influence of specific SNP loci on the outcomes.

All two-sided analyses were conducted using R software (version 4.3.2) and the R packages “TwoSampleMR,” “MRPRESSO” and “MVMR.”

## Results

### Univariable MR estimates and sensitivity analyses

**Figure 2:**
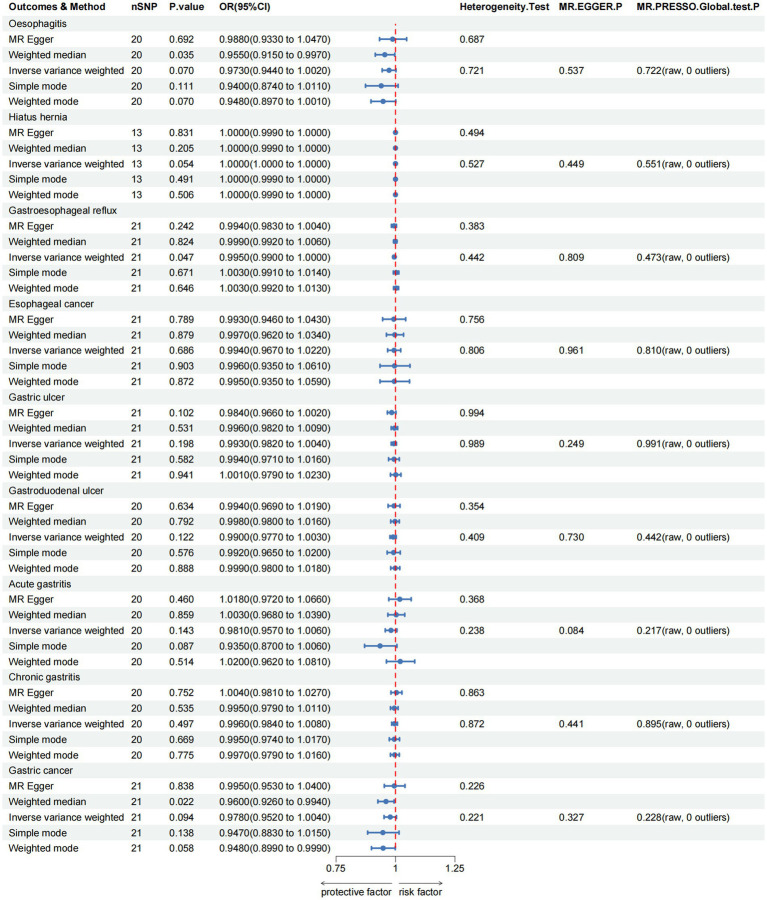
MR analysis of green tea intake to upper gastrointestinal diseases in the European population. OR, odds ratio; CI, confidence interval; MR, Mendelian randomization.

For the upper gastrointestinal diseases, as depicted in Figure 2 and Supplementary Table S4, our findings show a suggestive causal relationship between green tea intake and gastroesophageal reflux (OR = 0.9950, 95% CI 0.9900–1.0000, *p*_IVW_ = 0.047) based on the IVW analysis. However, the analysis of other upper gastrointestinal disorders did not yield statistically significant results (*p*_IVW_ > 0.05).

**Figure 3:**
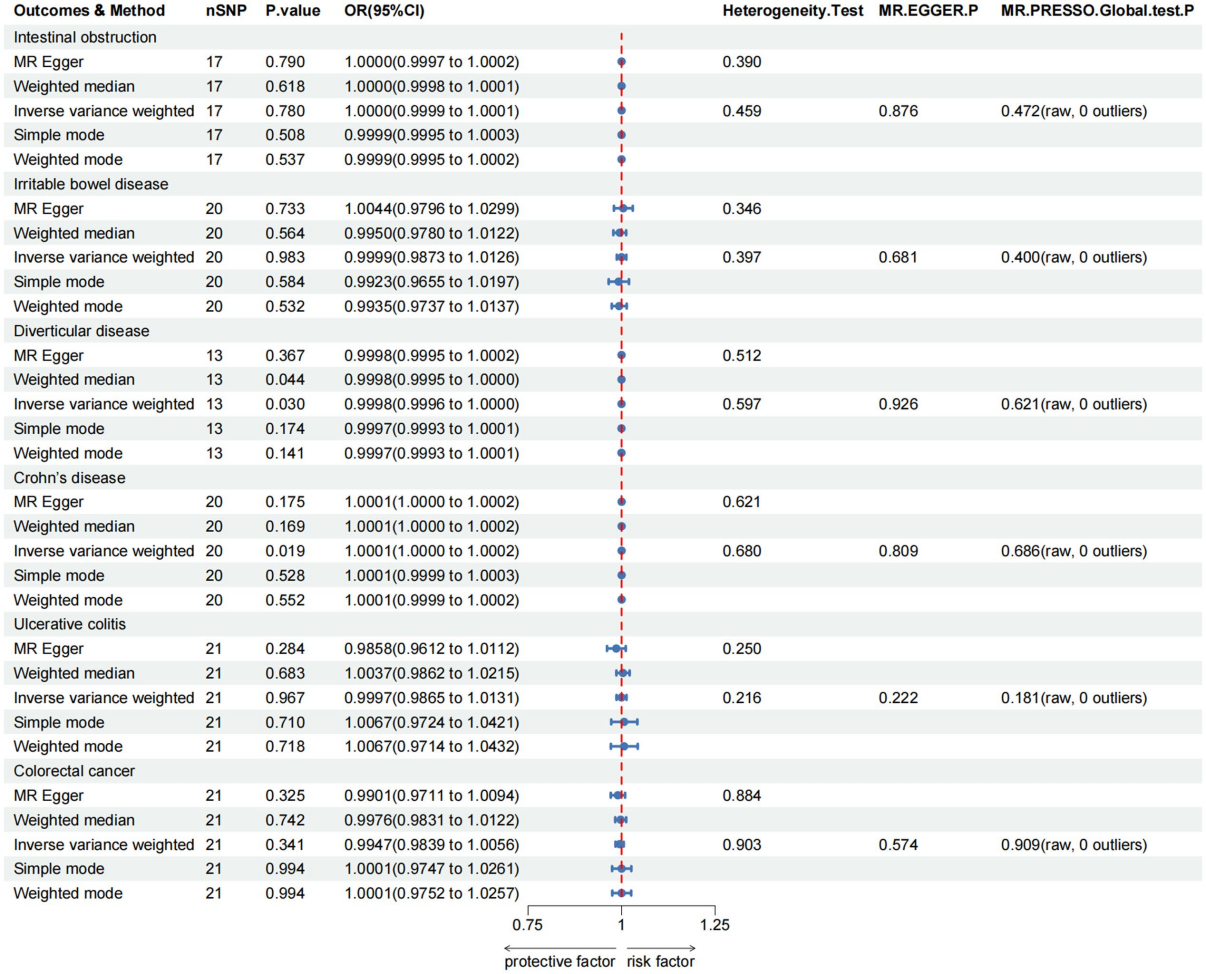
MR analysis of green tea intake to lower gastrointestinal diseases in the European population. OR, odds ratio; CI, confidence interval; MR, Mendelian randomization.

For the lower gastrointestinal diseases, as illustrated in Figure 3 and Supplementary Table S4, this study reveals greater green tea intake might suggestively decrease the risk of diverticulosis (OR = 0.9998, 95% CI 0.9996–1.0000, *p*_IVW_ = 0.030), while suggestively increase the risk of Crohn’s disease (OR = 1.0001, 95% CI 1.0000–1.0002, *p*_IVW_ = 0.019). However, in the analysis of other lower gastrointestinal diseases, the IVW results did not achieve statistical significance (*p*_IVW_ > 0.05).

**Figure 4:**
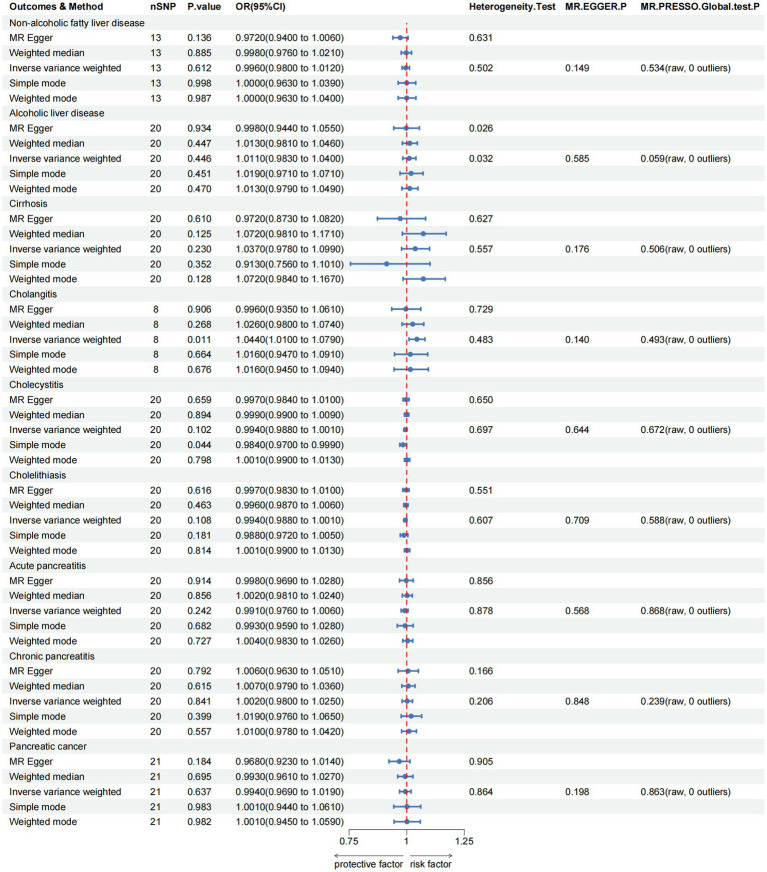
MR analysis of green tea intake to hepatobiliary and pancreatic diseases in the European population. OR, odds ratio; CI, confidence interval; MR, Mendelian randomization.

For the hepatobiliary and pancreatic diseases, a suggestive negative causal relationship between green tea intake and cholangitis was observed (OR = 1.0440, 95% CI 1.0100–1.0790, *p*_IVW_ = 0.011; Figure 4 and Supplementary Table S4). Additionally, our MR analyses revealed no significant association between green tea intake and the other hepatobiliary and pancreatic diseases (*p*_IVW_ > 0.05).

**Figure 5:**
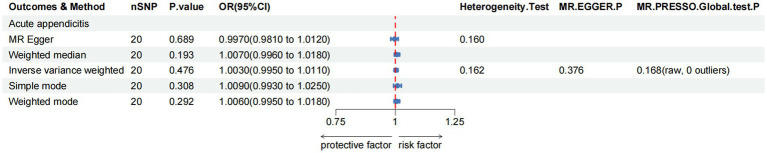
MR analysis of green tea intake to acute appendicitis in the European population. OR, odds ratio; CI, confidence interval; MR, Mendelian randomization.

For the acute appendicitis, the primary results of IVW revealed that greater green tea consumption was not significantly associated with the risk of acute appendicitis (*p*_IVW_ > 0.05; Figure 5 and Supplementary Table S4).

**Figure 6:**
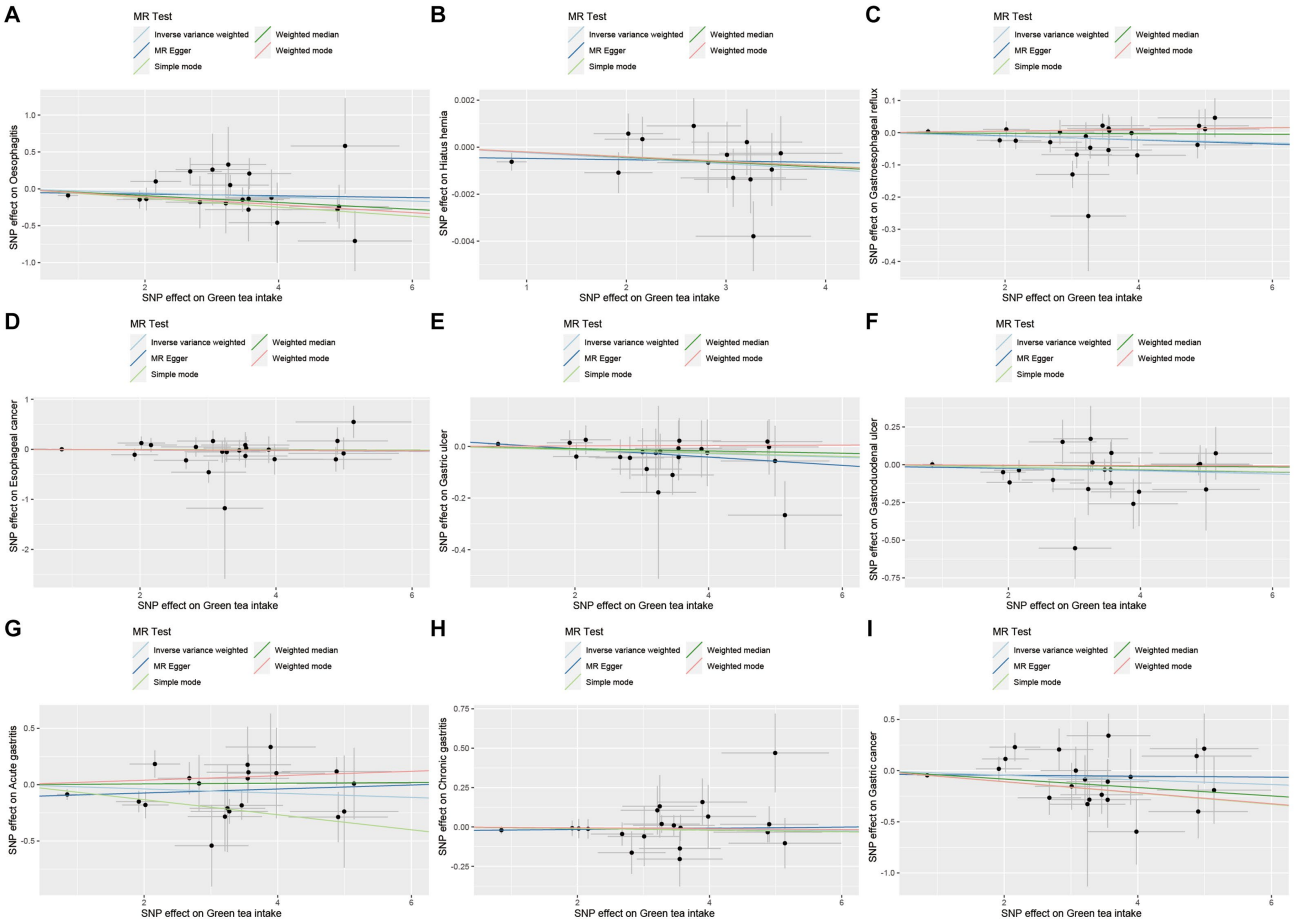
Scatter plot of SNPs associated with green tea intake on upper gastrointestinal diseases. **(A)** Oesophagitis. **(B)** Hiatus hernia. **(C)** Gastroesophageal reflux. **(D)** Esophageal cancer. **(E)** Gastric ulcer. **(F)** Gastroduodenal ulcer. **(G)** Acute gastritis. **(H)** Chronic gastritis. **(I)** Gastric cancer.

**Figure 7:**
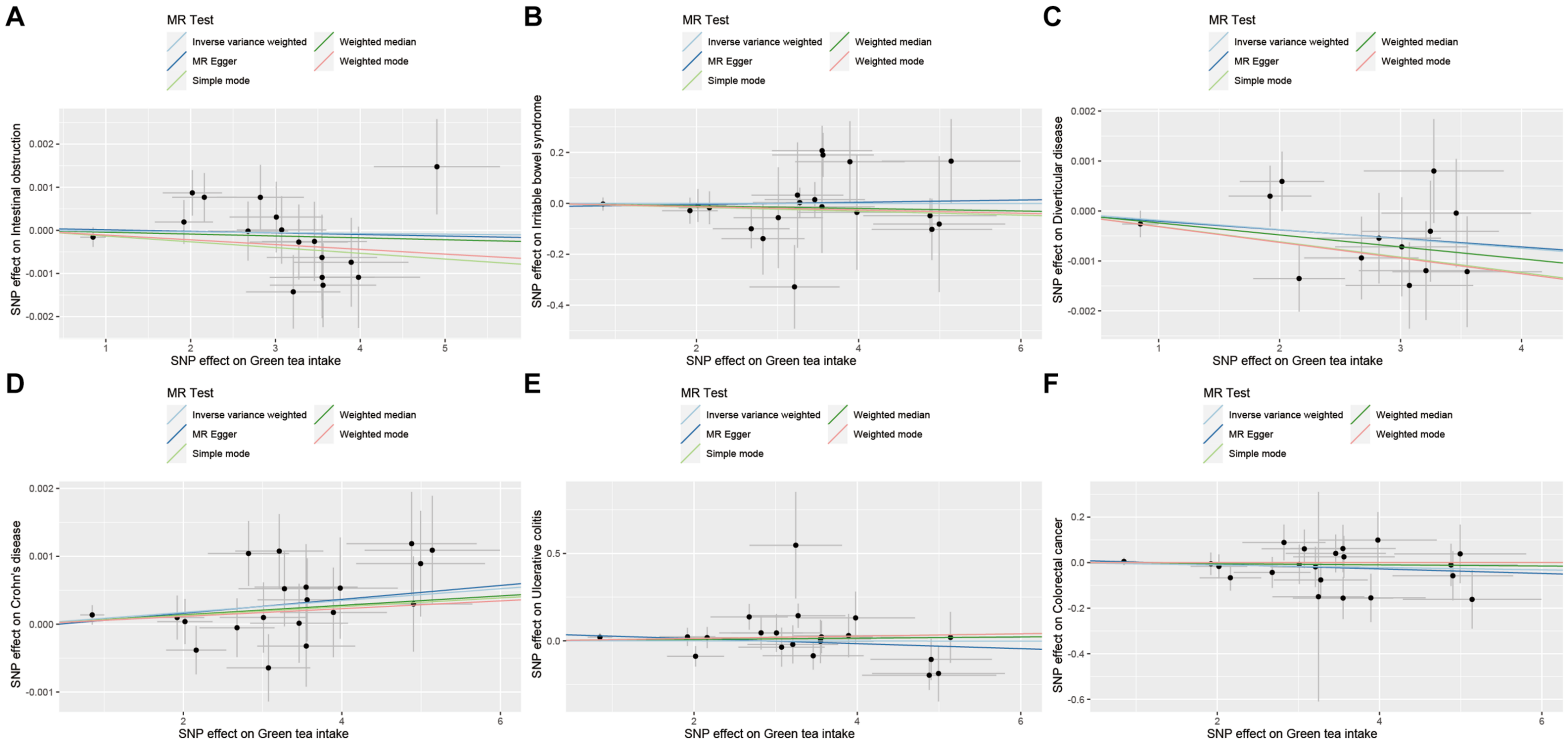
Scatter plot of SNPs associated with green tea intake on lower gastrointestinal diseases. **(A)** Intestinal obstruction. **(B)** Irritable bowel disease. **(C)** Diverticular disease. **(D)** Crohn’s disease. **(E)** Ulcerative colitis. **(F)** Colorectal cancer.

**Figure 8:**
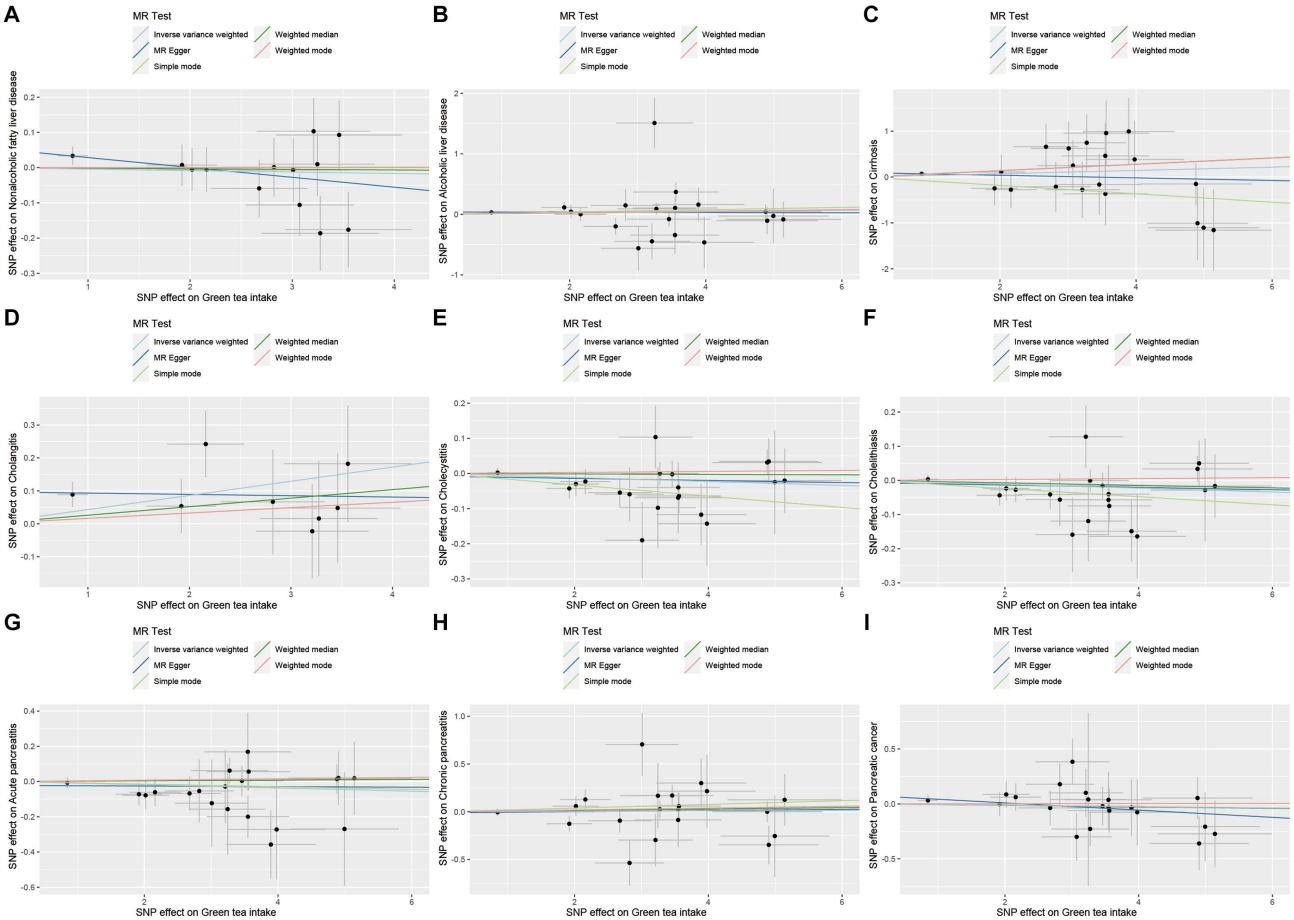
Scatter plot of SNPs associated with green tea intake on hepatobiliary and pancreatic diseases. **(A)** Non-alcoholic fatty liver disease. **(B)** Alcoholic liver disease. **(C)** Cirrhosis. **(D)** Cholangitis. **(E)** Cholecystitis. **(F)** Cholelithiasis. **(G)** Acute pancreatitis. **(H)** Chronic pancreatitis. **(I)** Pancreatic cancer.

**Figure 9:**
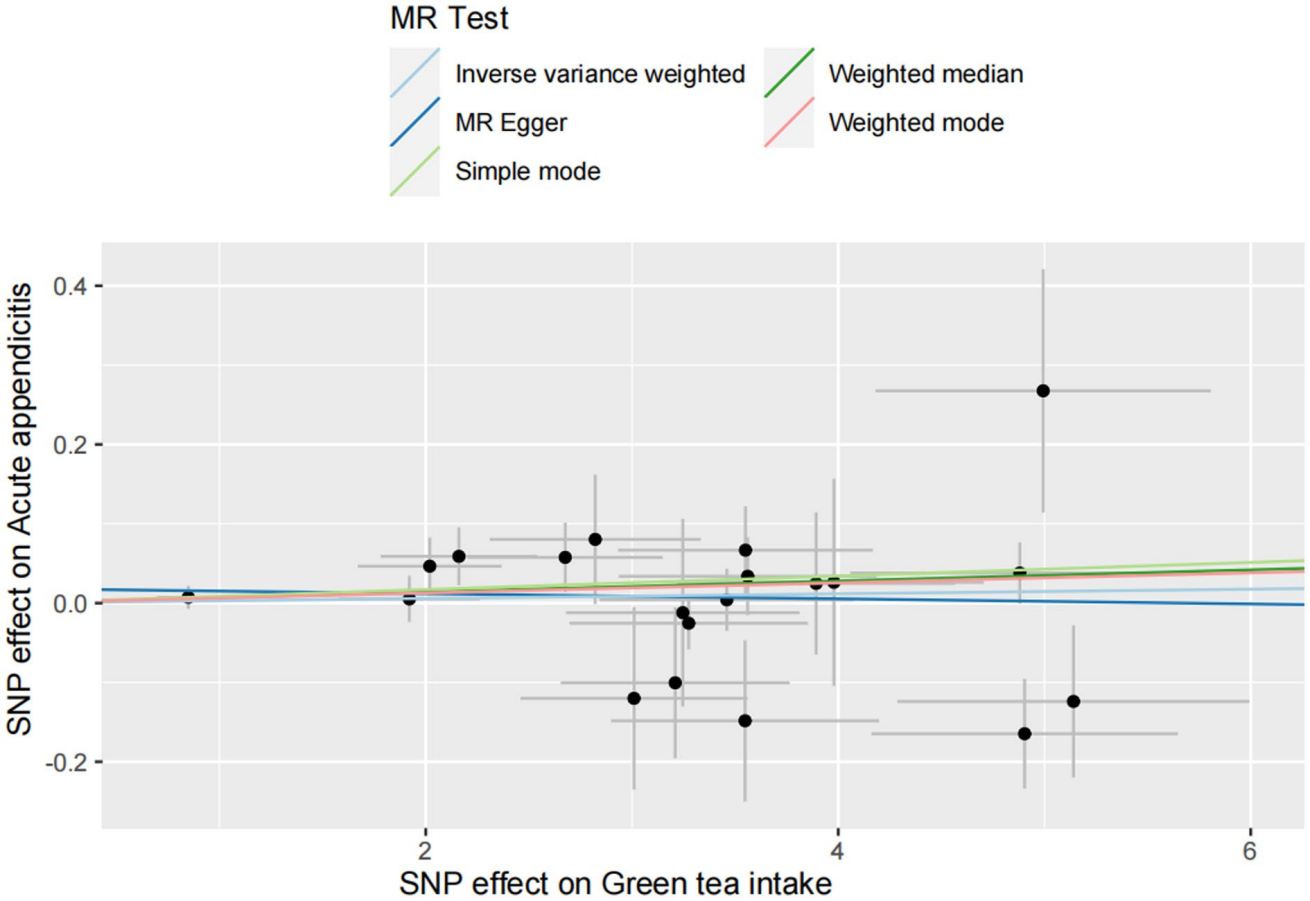
Scatter plot of SNPs associated with green tea intake on acute appendicitis.

As demonstrated in Figures 6–9, this study presents MR regression slopes and individual causal estimates for green tea intake concerning each SNP locus corresponding to upper gastrointestinal disease, lower gastrointestinal disease, hepatobiliary and pancreatic disease, and acute appendicitis. Supplementary Table S5 reveals that Cochrane *Q*-tests indicated no significant heterogeneity between the investigated outcomes and exposures (*p*_Cochrane *Q*_ > 0.05), except for heterogeneity observed between green tea intake and alcoholic liver disease (*p*_Cochrane *Q*_ < 0.05). In cases of heterogeneity, a random-effects model was applied to estimate the MR effect sizes, and no adjustments were deemed necessary for the study results due to heterogeneity. Moreover, the results of the MR-Egger and MR-PRESSO totality tests revealed a symmetric funnel plot (Supplementary Figure S2) and indicated no significant horizontal pleiotropy among any of the examined outcomes and exposures (*p* > 0.05).

### Multivariate MR estimates and sensitivity analyses

After controlling for potential confounders such as smoking and alcohol consumption, multivariate MR analysis showed that greater green tea consumption in European population was suggestively associated with the decreased risk of oesophagitis (OR = 0.9667, 95% CI: 0.9405–0.9936, *p*_IVW_ = 0.016) and gastric cancer (OR = 0.9810, 95% CI: 0.9628–0.9996, *p*_IVW_ = 0.046). Nevertheless, multivariate MR analysis also showed that greater green tea consumption was suggestively associated with the increased risk of Crohn’s disease (OR = 1.0001, 95% CI: 1.0000–1.0002, *p*_IVW_ = 0.007). However, the IVW results showed that green tea intake was not associated with the risk of other gastrointestinal disorders (*p*_IVW_ > 0.05), as shown in Supplementary Table S6.

In the sensitivity analysis, Cochrane’s *Q* test indicated the most MR analyses did not have heterogeneous (*p*_Cochrane *Q*_ > 0.05). Even in cases where heterogeneity was observed, its impact on our results was small due to our use of the IVW method. Meanwhile, according to the MR-Egger intercept, we found no evidence of horizontal pleiotropy in most association (*p*_intercept_ > 0.05; Supplementary Table S6).

## Discussion

Numerous studies have indicated a potential link between green tea consumption and gastrointestinal disorders. However, this association remains incompletely understood. Therefore, the current study employed a two-sample Mendelian randomization approach to elucidate the causal relationship between green tea consumption and these disorders. The IVW analysis revealed green tea consumption was suggestively associated with the decreased risk of oesophagitis and gastric cancer, alongside associated with the increased risk of Crohn’s disease. These findings offer valuable dietary insights for both the treatment and prevention of gastrointestinal ailments.

MR has been employed to investigate the association between green tea consumption and gastrointestinal diseases. An MR analysis focused on an East Asian population revealed that increased tea consumption was associated with a reduced risk of gastric cancer (OR = 0.90, 95% CI 0.82–0.99, *p* = 0.037) (31) which is consistent with our findings. However, some studies have reported no significant association between green tea consumption and the risk of gastric cancer (32), likely attributed to differences in the samples used in our study. Future studies will require larger GWAS databases to confirm this relationship.

Gastric cancer (GC) is the fifth most common cancer and the third most common cause of cancer deaths worldwide, and its main risk factors include bacterial infections, dietary influences, and age factors (33). Green tea and its main component, catechins, are believed to have some cancer-preventive effects (34) and are able to exert anticancer functions by inhibiting cell proliferation (35). However, the effect of green tea intake on gastric cancer risk remains a controversial topic. Our results suggest that there may be a negative causal relationship between green tea intake and gastric cancer in upper gastrointestinal diseases. This result is consistent with the conclusion reached in a Mendelian randomization study, which suggested that green tea consumption may be a potential protective factor against gastric cancer (31). However, the results of another review failed to confirm the association between tea consumption and gastric cancer (36). The discrepancy between our findings and those of the aforementioned study may stem from confounding factors and reverse causality present in previous studies, and thus further investigation is needed to confirm the association. In addition, since this study operated at the gene level through SNP loci, there were discrepancies between the observed results and real-world test scenarios. Therefore, although our findings provide potential insights for adjusting the dietary patterns of gastric cancer patients, they need to be interpreted with caution. In order to more clearly define the causal relationship between green tea intake and gastric cancer, future studies require more refined study designs and large-scale epidemiologic investigations. These efforts are essential to establish a reliable understanding of the relationship between green tea intake and gastric cancer.

Inflammation and damage to the mucosa of the esophagus known as oesophagitis is common worldwide and has a major impact on health and quality of life. A variety of physical, chemical and infectious agents can cause oesophagitis (37). Although studies exploring the correlation between green tea consumption and oesophagitis were lacking within our search, we delved into this relationship through a two-sample MR analysis. Our study revealed a suggestive causal relationship between green tea intake and oesophagitis. Thus, our exploration of the relationship between green tea consumption and oesophagitis could provide valuable insights into nutritional recommendations and disease management, potentially enhancing clinical approaches to managing this disease.

Epidemiological studies present conflicting findings regarding the correlation between beverage consumption, including green tea intake, and the risk of Crohn’s disease (14). Among dietary investigations in the clinical realm of Crohn’s disease, analyses concentrating on individual nutrients or foods prevail. The stimulative impact of green tea’s caffeine content on gastrointestinal motility could potentially exacerbate Crohn’s disease symptoms by elevating bowel movement frequency (8). Our findings uncovered a potential positive causal link between tea intake and Crohn’s disease. The outcomes of a Mendelian analysis suggest that genetically induced exposure to tea intake elevates the susceptibility to Crohn’s disease (38). Nevertheless, contrasting findings emerged from another meta-analysis, indicating a negative correlation between tea intake and Crohn’s disease risk (14). However, some studies have failed to yield significant evidence supporting an association between tea intake and Crohn’s disease (13). Hence, there could be merit in instituting surveillance and preventive measures for Crohn’s disease within populations exhibiting heightened tea intake.

We also investigated the association between green tea intake and other gastrointestinal disorders. However, the results of our two-sample Mendelian randomization analysis indicated no significant relationship (*p*_IVW_ > 0.05). Furthermore, in a sizable community-based prospective cohort study, green tea consumption did not correlate with the risk of gastric cancer (39). Moreover, substantial evidence supporting an association between tea intake and ulcerative colitis or non-alcoholic fatty liver disease is reportedly lacking (13). Additionally, one study found no overall significant association between green tea consumption and the risk of colorectal cancer (40). The outcomes of another prospective cohort study’s meta-analysis similarly indicated no significant impact of tea consumption on colorectal cancer risk among both men and women (41). Nevertheless, several studies diverge from our findings. Green tea has been identified as a protective agent against gastric cancer (15, 42) and colorectal cancer (16, 43). Moreover, in a large prospective cohort study, moderate tea consumption (0.5–1 cup/day) correlated with a reduced risk of irritable bowel syndrome (44). Additionally, a case-control study revealed that frequent tea consumption was associated with decreased odds of ulcerative colitis, even after adjusting for age, sex, body mass index, and smoking history (45). Furthermore, A small increase in the intake of flavan-3-ol-rich foods (e.g., green tea) may help improve the survival of colorectal cancer patients (46). The reason this is different from our findings may be that we limited the type of tea ingested to green tea, and thus our results do not make sense. Alternatively, it could be attributed to heterogeneity between the studies, warranting further experimental design to elucidate the relationship.

The relationship between green tea and gastrointestinal disorders can be explained by a variety of biological mechanisms. Chalazonitis et al. (47) showed that brain dopamine neurotrophic factor is essential for the development, maintenance and gastrointestinal transit regulation of intestinal neurons, which is in line with the results of the present study. In lower gastrointestinal diseases, for colitis, a study by Belle et al. (48) demonstrated that TFF3 interacts with LINGO2 to regulate EGFR activation and is able to prevent colitis. For colon cancer, a study by Aljahdali et al. (49) found a strong association between IPO11 downregulation and poorer colon cancer patient survival. And the co-expression gene profile of IPO11 was similarly associated with colon carcinogenesis. Similarly, Yang et al. (50) showed that FITM2 in colon adenocarcinoma has been shown to be a key mRNA. a study by D’Ermo et al. (51) showed that pathogenic mutations in the PTEN oncogene lead to continuous involvement of the gastrointestinal tract and eventually to the development of diseases such as colorectal cancer. In hepatobiliary and pancreatic diseases, for nonalcoholic fatty liver disease, Li et al. (52) found that vascular endothelial growth factor B (VEGFB) has the potential to regulate lipid metabolism and plays a regulatory role in the development and progression of NAFLD. For pancreatic cancer, Rizzato et al. (53) showed that the CTNNA2 gene was associated with survival in patients with pancreatic ductal adenocarcinoma. These studies support our results, as the above genes are the nearest gene of SNPs associated with green tea intake.

Ohishi et al. (54) showed that green tea and EGCG inhibited gene expression of inflammation-related enzymes and had beneficial effects on inflammatory diseases. Since green tea and EGCG have multiple targets and act as anti-inflammatory agents in a pleiotropic manner, including acting as an antioxidant scavenging mechanism for reactive oxygen species, leading to attenuation of nuclear factor-κB activity. A study by Kim et al. (55) showed that a combined extract of *Artemisia absinthium* and green tea (MPGT) had significant antioxidant or anti-inflammatory effects on *Helicobacter pylori*-associated gastropathy. Among them, the significant protective changes in alcoholic gastritis with MPGT were associated with increased expression of cytoprotective genes such as heat shock protein (HSP) 27, HSP60 and PDGF, and significant reduction in the levels of calcium-dependent phospholipase A2, MAPK and NF-κB. Therefore, we may consider the use of EGCG-rich green tea to improve the quality of life of patients with gastrointestinal diseases (56).

This study boasts several strengths. Firstly, to our knowledge, our study is the first comprehensive exploration of the causal relationship between green tea intake and various gastrointestinal disorders, including upper gastrointestinal diseases, lower gastrointestinal diseases, hepatobiliary and pancreatic diseases, and acute appendicitis. Second, it delves into the correlation between green tea intake and gastrointestinal diseases at a genetic level, thereby mitigating confounding factors and biases, thus significantly bolstering the precision and credibility of the findings. Secondly, the study employs chained unbalanced clustering, heterogeneity detection, horizontal pleiotropy detection, and leave-one-out techniques to conduct a sensitivity analysis of the data, further solidifying the stability of the causal relationship.

Nonetheless, this study is not without its limitations, which could account for the variance between its findings and those of existing literature. Firstly, this study focuses on the European population; however, green tea consumption in Europe is relatively low. According to the Tea Council of Great Britain, black tea imports into the UK in 2023 will be £226 million and green tea imports will be £22.62 million. Thus limiting the generalizability of the results of this study. Second, the study included only 122 cirrhosis patients, which may affect the robustness of the conclusions drawn. Thirdly, our pooled data on green tea consumption were obtained from the UK Biobank database with data from 24-h dietary recalls, which may be subject to recall bias to the extent of attenuating the true association, and there is a lack of SNP data on green tea frequency, the exposure variable in this study may not be representative of green tea intake in the long term. Fourthly, the exposure data and partial outcome data were obtained from the UKB database, potentially introducing bias due to sample overlap. Fifthly, we utilized the LDtrait tool to find the SNP identified in our study were confounders, which utilizes GWAS summary statistics gathered from the GWAS Catalog, which may result in the searches may not be comprehensive. Consequently, future research endeavors should strive to address these issues to attain a more comprehensive understanding of the relationship between green tea intake and gastrointestinal disorders.

## Conclusion

Our two-sample MR analysis suggests greater green tea consumption was suggestively associated with the decreased risk of oesophagitis and gastric cancer, while greater green tea consumption in was suggestively associated with the increased risk of Crohn’s disease. Moreover, larger GWAS databases will be needed in the future to confirm this relationship to ensure the robustness and reliability of the study results.

## Data Availability

The original contributions presented in the study are included in the article/Supplementary material, further inquiries can be directed to the corresponding authors.

## Footnotes

^1^https://www.ebi.ac.uk/gwas/

^2^https://ldlink.nih.gov/?tab=ldtrait/
