# Supplementary material for: Causal relationship between green tea intake and gastrointestinal disorders: a two-sample Mendelian randomization study
Source: Front Nutr. 2024 Sep 20;11:1426779. doi: 10.3389/fnut.2024.1426779 (PMC11449853; doi:10.3389/fnut.2024.1426779)
Supplement: Supplementary file 2 [file Image_1.pdf]

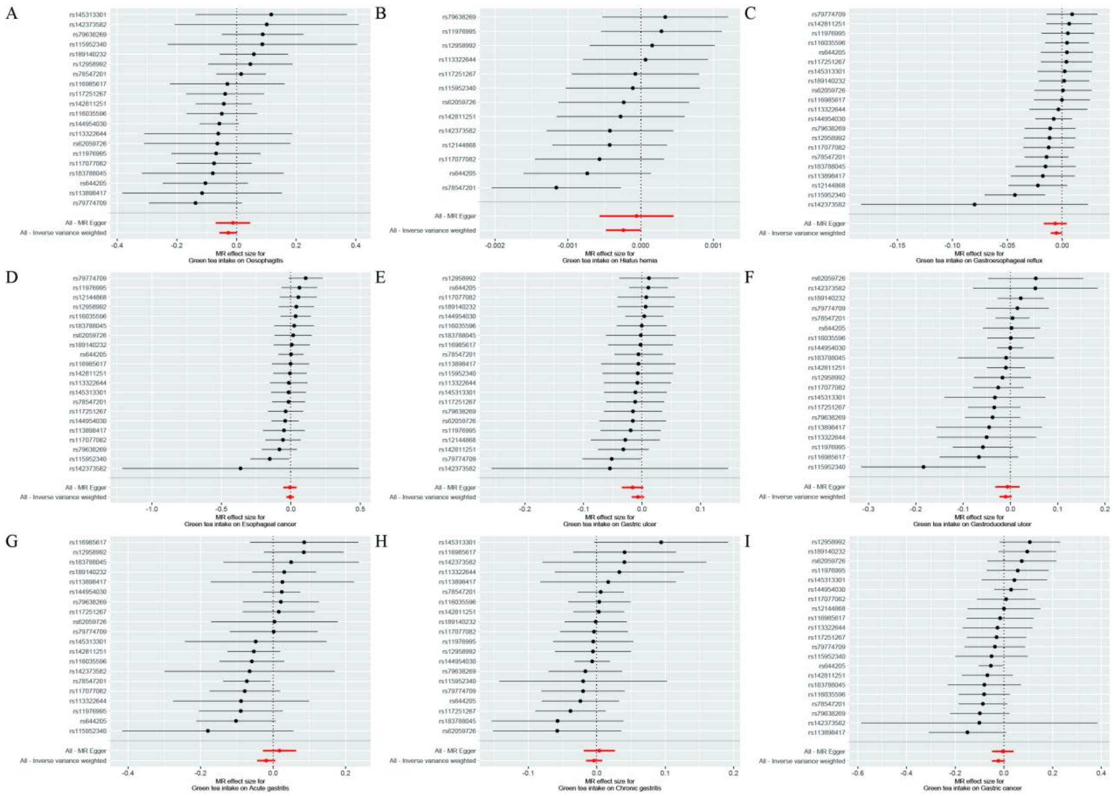

Figure S1. Forest plot of SNPs associated with green tea intake on upper gastrointestinal diseases. A. Oesophagitis; B. Hiatus hernia; C. Gastroesophageal reflux; D. Esophageal cancer; E. Gastric ulcer; F. Gastroduodenal ulcer; G. Acute gastritis; H. Chronic gastritis; I. Gastric cancer.

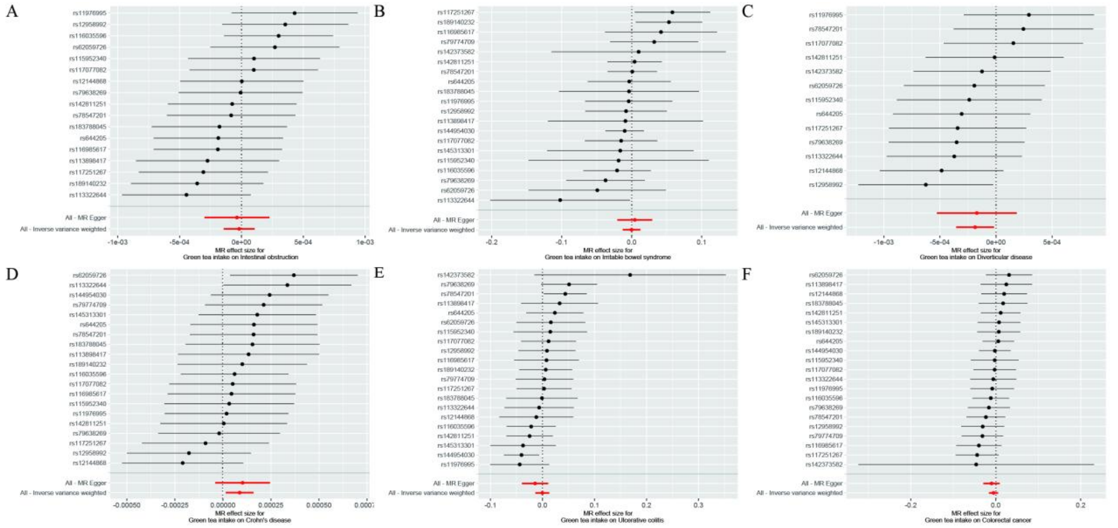

Figure S2. Forest plot of SNPs associated with green tea intake on lower gastrointestinal diseases. A. Intestinal obstruction; B. Irritable bowel disease; C. Diverticular disease; D. Crohn ’ s disease; E. Ulcerative colitis; F. Colorectal cancer.

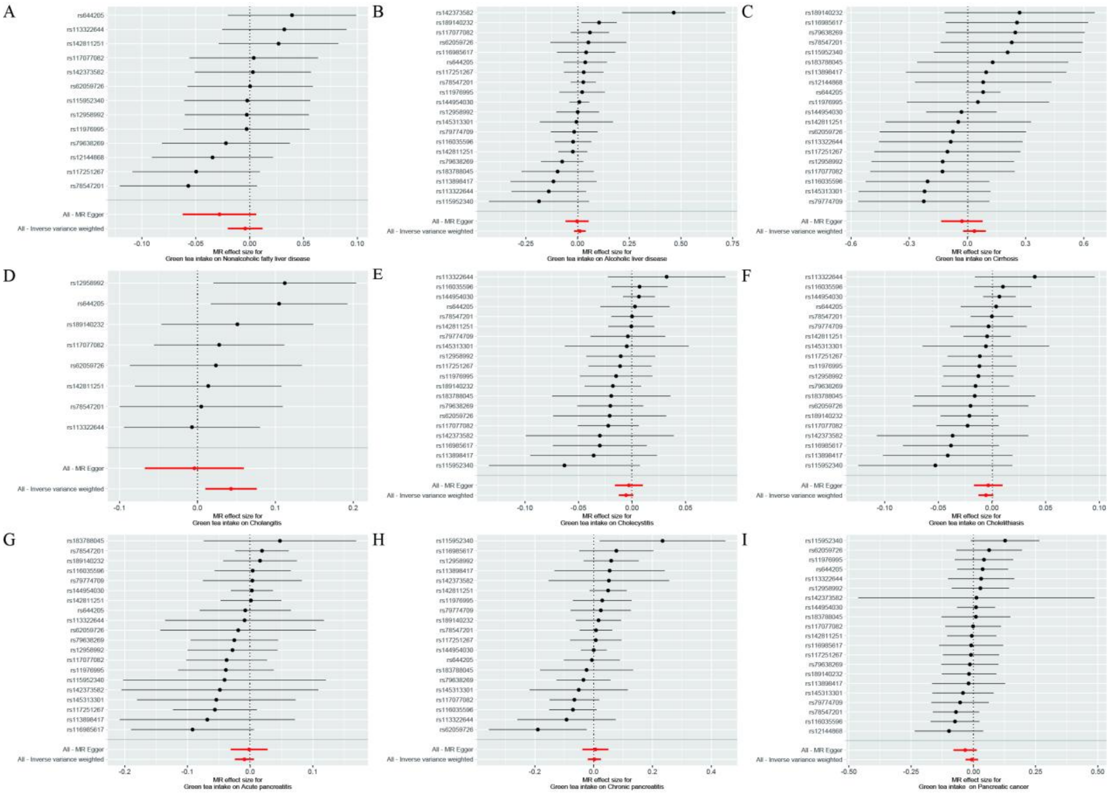

Figure S3. Forest plot of SNPs associated with green tea intake on hepatobiliary and pancreatic diseases. A. Non-alcoholic fatty liver disease; B. Alcoholic liver disease; C. Cirrhosis; D. Cholangitis; E. Cholecystitis; F. Cholelithiasis; G. Acute pancreatitis; H. Chronic pancreatitis; I. Pancreatic cancer.

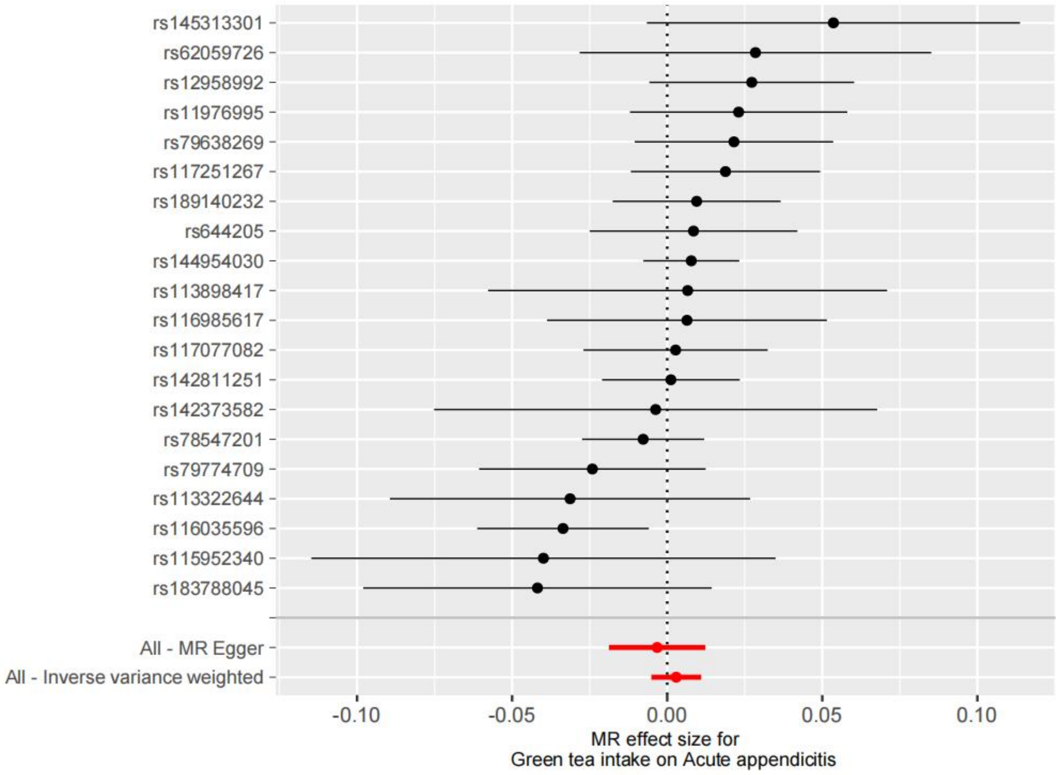

Figure S4. Forest plot of SNPs associated with green tea intake on acute appendicitis.

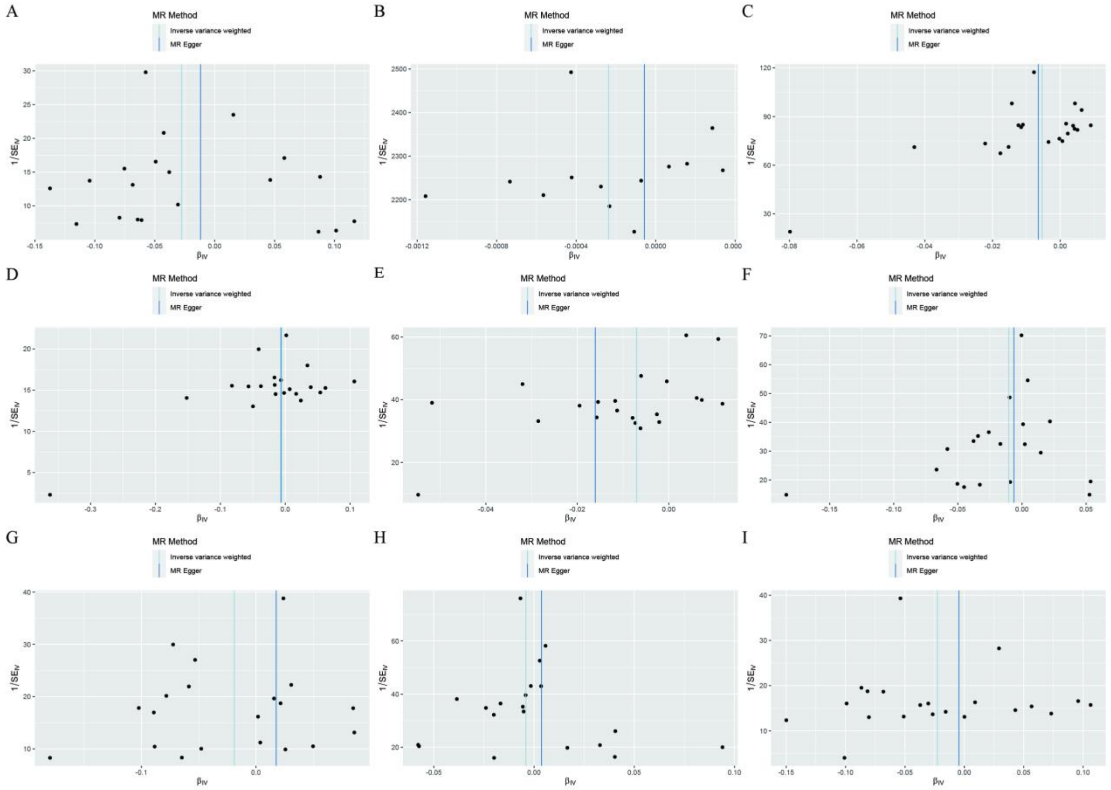

Figure S5. Funnel plot of SNPs associated with green tea intake on upper gastrointestinal diseases. A. Oesophagitis; B. Hiatus hernia; C. Gastroesophageal reflux; D. Esophageal cancer; E. Gastric ulcer; F. Gastroduodenal ulcer; G. Acute gastritis; H. Chronic gastritis; I. Gastric cancer.

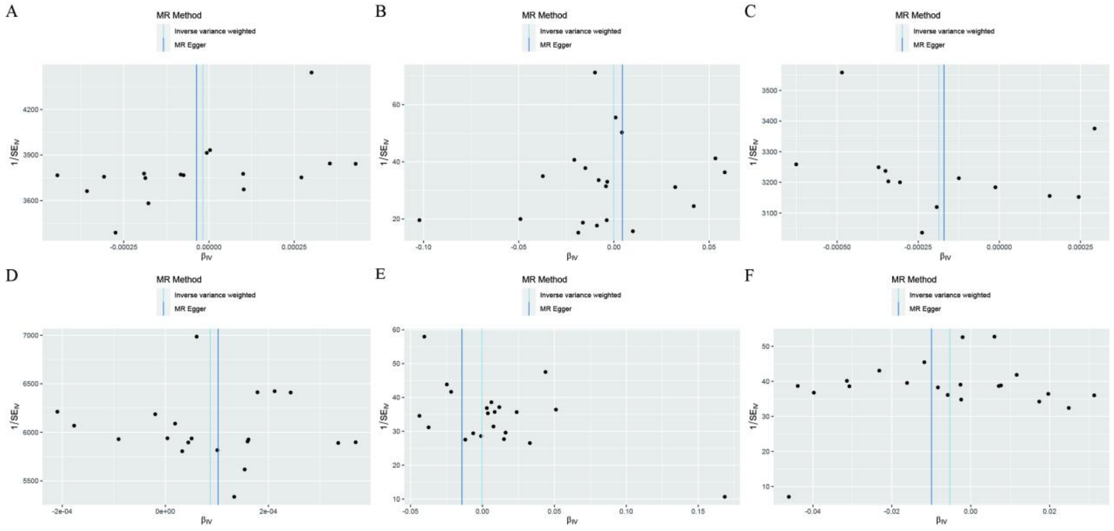

Figure S6. Funnel plot of SNPs associated with green tea intake on lower gastrointestinal diseases. A. Intestinal obstruction; B. Irritable bowel disease; C. Diverticular disease; D. Crohn ’ s disease; E. Ulcerative colitis; F. Colorectal cancer.

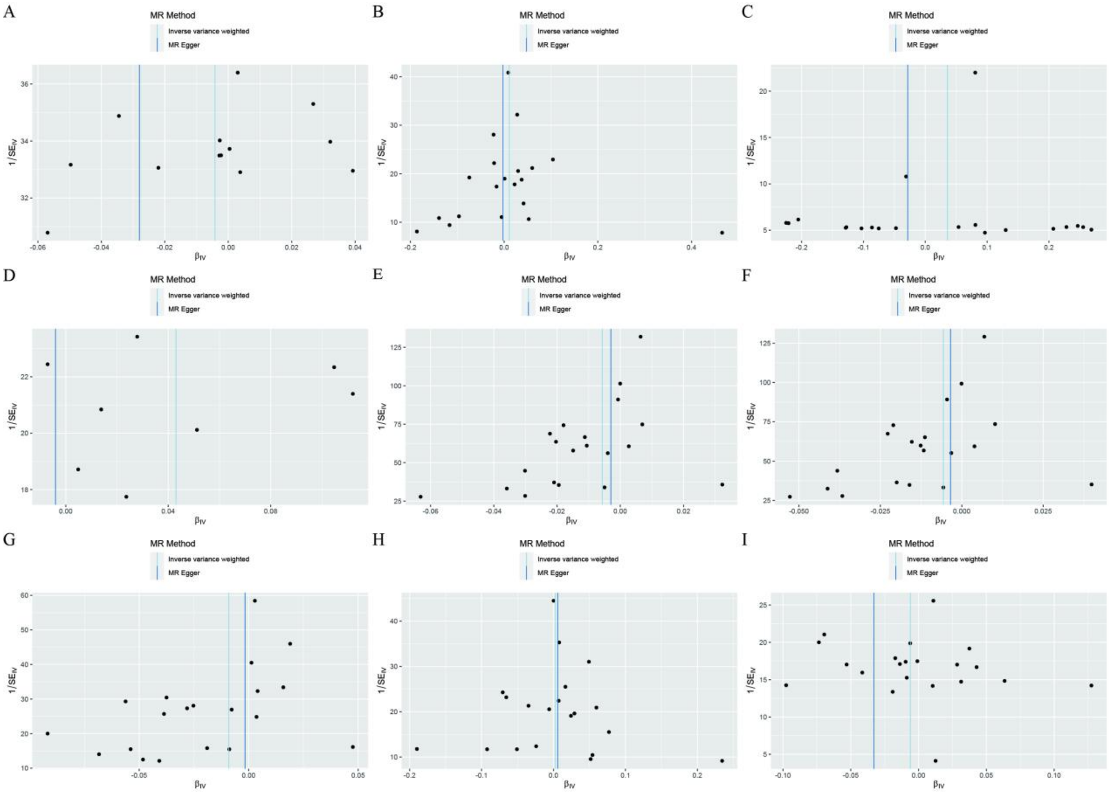

Figure S7. Funnel plot of SNPs associated with green tea intake on hepatobiliary and pancreatic diseases. A. Non-alcoholic fatty liver disease; B. Alcoholic liver disease; C. Cirrhosis; D. Cholangitis; E. Cholecystitis; F. Cholelithiasis; G. Acute pancreatitis; H. Chronic pancreatitis; I. Pancreatic cancer.

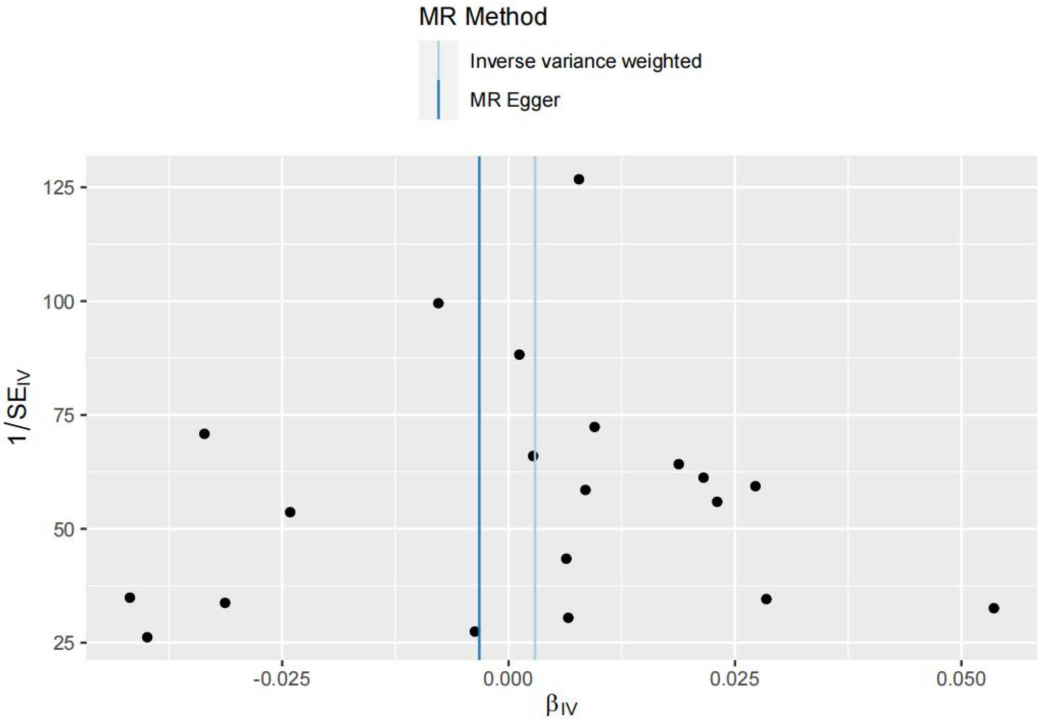

Figure S8. Funnel plot of SNPs associated with green tea intake on acute appendicitis.
